# Risk Areas for Influenza A(H5) Environmental Contamination in Live Bird Markets, Dhaka, Bangladesh

**DOI:** 10.3201/eid2709.204447

**Published:** 2021-09

**Authors:** Shovon Chakma, Muzaffar G. Osmani, Holy Akwar, Zakiul Hasan, Tanzinah Nasrin, Md Rezaul Karim, Mohammed Abdus Samad, Mohammad Giasuddin, Peter Sly, Zahir Islam, Nitish Chandra Debnath, Eric Brum, Ricardo Soares Magalhães

**Affiliations:** Emergency Centre for Transboundary Animal Diseases, Food and Agriculture Organization of the United Nations, Dhaka, Bangladesh (S. Chakma, H. Akwar, Z. Hasan, T. Nasrin, N.C. Debnath, E. Brum);; The University of Queensland, Brisbane, Queensland, Australia (S. Chakma, P. Sly, Z. Islam, R. Soares Magalhães);; Department of Livestock Services, Dhaka (M.G. Osmani);; Bangladesh Livestock Research Institute, Savar, Bangladesh (M. Rezaul Karim, M. Abdus Samad, M. Giasuddin)

**Keywords:** influenza A(H5) virus, live bird markets, environmental contamination, risk factors, influenza, zoonoses, Dhaka, Bangladesh, viruses

## Abstract

We evaluated the presence of influenza A(H5) virus environmental contamination in live bird markets (LBMs) in Dhaka, Bangladesh. By using Bernoulli generalized linear models and multinomial logistic regression models, we quantified LBM-level factors associated with market work zone–specific influenza A(H5) virus contamination patterns. Results showed higher environmental contamination in LBMs that have wholesale and retail operations compared with retail-only markets (relative risk 0.69, 95% 0.51–0.93; p = 0.012) and in March compared with January (relative risk 2.07, 95% CI 1.44–2.96; p<0.001). Influenza A(H5) environmental contamination remains a public health problem in most LBMs in Dhaka, which underscores the need to implement enhanced biosecurity interventions in LBMs in Bangladesh.

Live bird markets (LBMs) have long been identified as major sites for the maintenance, transmission, amplification, and dissemination of influenza A(H5) virus (*1*,*2*). Studies in the United States, China, Indonesia, and Vietnam have shown that LBMs can pose a public health risk for zoonotic spill-over to humans through environmental contamination (*2*–*8*). In Bangladesh, the first evidence of zoonotic transmission of influenza A(H5) virus emerged in 2012; LBMs in Dhaka were considered the main source of exposure for all 3 human cases reported (*9*,*10*). The relatively low level of influenza A(H5) endemicity found in studies conducted in LBMs in Bangladesh since 2012 (e.g., <10% prevalence at live bird sampling level) (*11*–*13*) have contributed to a false sense of security regarding contamination risk. Indeed, since 2013, several influenza A(H5) outbreaks in poultry (9 outbreaks), wild birds (5 outbreaks), and humans (2 outbreaks) have occurred in Bangladesh (*14*,*15*). During March 2007–December 2020, Bangladesh reported 556 outbreaks of influenza A(H5) virus in poultry (*14*) and 8 cases in humans (*15*).

Environmental sampling in LBMs for the purposes of avian influenza virus surveillance was first introduced in the United States in 1986 (*16*). A recent study evaluated the effectiveness of environmental sampling for influenza A surveillance and described multiple sampling sites in an LBM (*17*). Earlier studies from Bangladesh primarily focused on collecting samples from market environment sites (such as market floor, stall floor, slaughter area, waste bin, poultry cage, water, fecal material on or underneath the poultry cage, blood, and poultry offal) to understand the LBM environment status for influenza A (*11*,*12*,*18*–*25*).

Few studies to date—1 in Indonesia and 3 in Guangdong, China—have performed simultaneous sampling in different LBM work zones, such as the poultry delivery, poultry holding, poultry slaughter, poultry sale, and waste disposal zones (*26*–*29*). These studies indicated that the poultry slaughter and sale zones were the 2 most contaminated LBM work zones for influenza A(H5N1) in Indonesia (*27*) and influenza A(H7N9), (H5), and (H9) in China (*26*,*28*,*29*). To date, no studies have been performed in Bangladesh on influenza A environmental contamination within different LBM work zones. The results from China and Indonesia have provided additional justification to evaluate the influenza A surveillance program of the Food and Agriculture Organization of the United Nations (FAO) in Bangladesh. Given the costs of maintaining influenza surveillance programs, epidemiologic evidence on within-market risk areas for contamination would help fine-tune current surveillance approaches in Bangladesh.

Implementing biosecurity practices in LBMs reduces environmental contamination with influenza A (*30*). For example, weekly market closures (>1 day) and everyday cleaning and disinfecting interventions were reported to reduce market contamination with avian influenza virus (H7N2) in the United States and influenza (H7N9) and (H9N2) in China (*5*,*31*,*32*). In Bangladesh, improved biosecurity practices at the market level have not effectively reduced environmental contamination for influenza A(H5) virus in Dhaka and Chittagong LBMs during 2012–2014 (*22*,*25*). Since 2014, no study has comprehensively reported the effect of market-level biosecurity practices on the probability of influenza A(H5) environmental contamination in Dhaka. Although the 2 administrative areas of the Dhaka metropolitan area (Dhaka North City Corporation [DNCC] and Dhaka South City Corporation [DSCC]) are known for their distinct demographic and urban features (*33*), no studies to date have investigated how biosecurity practices and influenza A(H5) contamination rates differ in relation to market-level characteristics of LBMs located in different parts of Dhaka. To inform the development of effective environmental sampling strategies for influenza surveillance in LBM, our study sought to characterize the differences in the proportion of influenza A(H5) environmental contamination in markets in DNCC and DSCC, to identify and quantify market-level factors associated with the probability of influenza A(H5) contamination in specific work zones (i.e., arrival, slaughtering and processing, and consumer exposure or sales), and to identify and quantify market-level factors associated with work zone–specific contamination patterns within LBMs.

## Materials and Methods

### Study Design for Influenza A(H5) Virus Surveillance in LBMs in Dhaka Metropolitan

**Figure 1:**
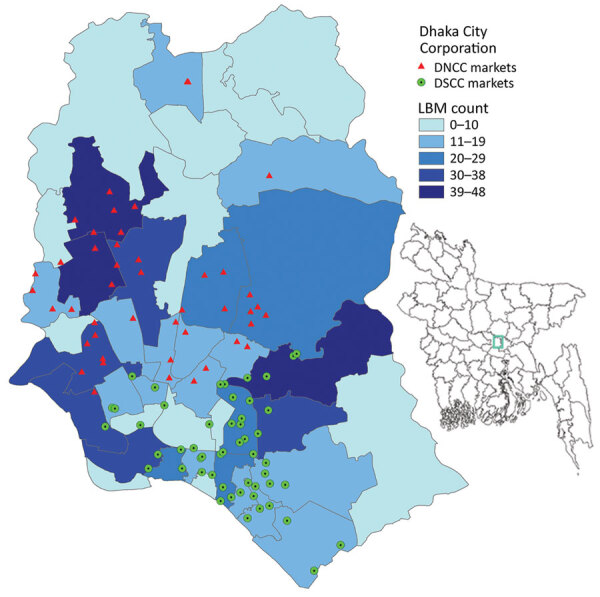
Locations of LBMs in the Dhaka metropolitan area, Bangladesh, January–March 2016. Inset map shows location of Dhaka in Bangladesh. DNCC, Dhaka North City Corporation; DSCC, Dhaka South City Corporation; LBM, live bird market.

We focused our investigation on the Dhaka metropolitan area, which has the highest population density (30,551 residents/km^2^) of all metropolitan areas in Bangladesh (*34*). We selected 104 LBMs within metropolitan Dhaka (Figure 1), which were part of the influenza surveillance initiative of the FAO and Department of Livestock Services (DLS) (Appendix) (*35*). Sampling targeted the months of January–March, which are known for a higher level of circulation of influenza A(H5) virus in poultry in Bangladesh (*36*).

We used data on market-level characteristics collected during the Dhaka LBM census to quantify the association between influenza A(H5) environmental contamination in LBMs and within specific market work zones adjusted for market-level characteristics (Appendix). Three market work zones (poultry arrival [A], poultry slaughtering and processing [S], and consumer exposure or sales [E]) and environmental sites in each work zone were selected for sampling on the basis of the findings from Indrani et al. (Appendix) (*27*).

### Collection, Preservation, and Transportation of Environmental Samples

Sample collectors from DLS, DNCC, and DSCC performed monthly collection of environmental samples from the selected LBMs. In a given visit, a pool of 6 samples were collected from each work zone using standard polyester-tipped swabs and stored separately in a 3 mL viral transport medium (Becton Dickinson, https://www.bd.com). Pooled samples were kept in ice boxes and transported to the DLS Central Disease Investigation Laboratory and Livestock Research Institute laboratory for temporary storage at 4°C. All samples were then transported in ice boxes to the National Reference Laboratory for Avian Influenza at Bangladesh Livestock Research Institute (Savar, Dhaka) and stored at −80°C before testing.

### Laboratory Testing

**Figure 2:**
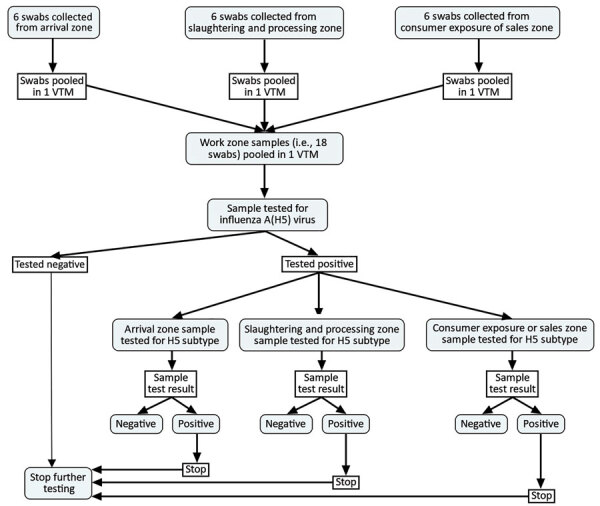
Sampling and laboratory testing protocol for influenza A(H5) in live bird markets, Dhaka, Bangladesh, January–March 2016. VTM, viral transport medium.

We tested for influenza A(H5) virus 18-swab pools from each selected market (i.e., 6 swabs/3 work zones) using real-time reverse transcription PCR (rRT-PCR). When an 18-swab pool of a market tested positive, further testing was carried out using rRT-PCR to confirm influenza A(H5) virus in the 6-swab pool of a specific work zone (Figure 2). We used MagMAX viral RNA isolation kit and KingFisher mL Purification System extractor (ThermoFisher Scientific, https://www.thermofisher.com) for RNA extraction. The rRT-PCR testing protocols followed the procedures recommended by the Australian Centre for Disease Preparedness quality assurance manual with influenza A(H5) primers (IVA D148 H5, IVA D149 H5, IVA D204f, and IVA D205r) and probes (IVA H5a and IVA D215P) produced at Australian Animal Health Laboratory and AgPath-ID One-Step RT-PCR Reagents (ThermoFisher Scientific). A pool sample was considered positive for influenza A(H5) if the cycle threshold value was <40 (*37*).

### Data Analyses

Our study included markets with information on both infection status and market-level characteristics (n = 97) and those with information on market-level infection status only (n = 7). In our analyses, we considered 2 outcomes of interest: presence or absence of influenza A(H5) virus environmental contamination in specific work zones and LBM–level zone-specific influenza A(H5) environmental contamination patterns. Work zone–specific environmental contamination patterns were classified as negative if all 3 work zones tested negative; ASE–positive when all 3 work zones tested positive; S only–positive when only the slaughtering and processing zone tested positive; SE– or AS–positive when the slaughtering and processing zone and 1 other work zone (E or A) tested positive; and other when the market tested positive for A only, E only, or both A and E.

We summarized DNCC and DSCC market-level biosecurity characteristics by using descriptive statistical analyses. Market-level biosecurity characteristics considered in the investigation included market location, market type, species sold, number of vendors, number of poultry species sold, dominant species (by comparing the poultry headcount), poultry headcount, electricity in the facility, presence of roof, running water in the facility, sale of poultry to other vendors, weekly market closure (>1 day), direct sale of poultry to consumers, sale of products other than poultry (i.e., fish, red meat, vegetables, groceries), daily cleaning protocol (at minimum with detergent), poultry slaughtering locations, and number of slaughtering facilities. We used a univariable Fisher exact test with a significance level of p<0.05 to identify differences in influenza A(H5) recovery by the geographic location of Dhaka markets. We then ran Bernoulli generalized linear models and multinomial logistic regression models to quantify risk factors associated with the probability of influenza A(H5) environmental contamination and work zone–specific contamination patterns (Appendix). The goodness-of-fit of the final multivariable model was assessed by Akaike information criterion (AIC), and the lowest AIC among all competing models was identified as the best fitting model in the study (*38*). We used Stata 15 (StataCorp LLC, https://www.stata.com) for statistical analyses.

## Results

### Characteristics of LBMs

Of 104 enrolled LBMs, a total of 97 markets (52 from DSCC and 45 from DNCC) had complete questionnaire information on their biosecurity characteristics (Appendix Table 1). The retail type of LBM was predominant in DSCC (84.62%, 45/52) and DNCC (64.44%, 29/45) of Dhaka. Most markets in DSCC (88.46%, 46/52) and DNCC (97.78%, 44/45) sold multiple species of poultry. The broiler chicken was the main species at LBMs in DSCC (69.23%, 36/52) and DNCC (80.00%, 36/45).

Market-level daily cleaning (at minimum with detergent) and weekly market closure (>1 day) practices varied among DNCC and DSCC markets. These 2 practices were reported to be more common in DSCC markets (75.00% [39/52] for daily cleaning and 45.15% [24/52] for weekly closure) compared with DNCC markets (31.11% [23/45] and 17.78% [8/45]). Most markets reported slaughtering poultry at vendor stalls (78.85% [41/52] in DSCC and 93.33% [42/45] in DNCC) (Appendix Table 1).

### Differences in the Proportion of Influenza A(H5) Virus Environmental Contamination and Market Characteristics

**Figure 3:**
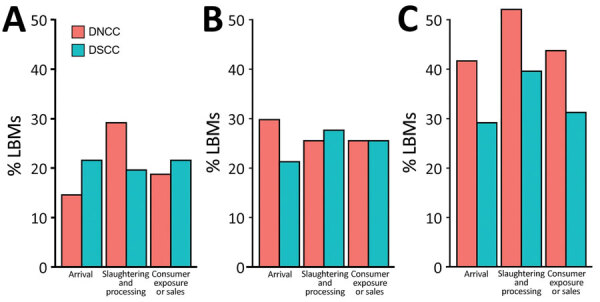
Distribution of influenza A(H5) virus environmental contamination in specific work zones in LBMs of DNCC and DSCC, Dhaka, Bangladesh, January–March 2016. A) January; B) February; C) March. DNCC, Dhaka North City Corporation; DSCC, Dhaka South City Corporation; LBM, live bird market.

Our analysis indicates that the proportion of influenza A(H5) virus environmental contamination was significantly higher in March than the previous 2 months (p≤0.001) (Appendix Table 2). The trend of LBM work zone–specific influenza A(H5) environmental contamination was similar in March in DSCC and DNCC markets, and the highest level of environmental contamination was in the slaughtering and processing zone (Figure 3). Of all market-level characteristics, only 3 characteristics were found to be significantly associated with proportions of influenza A(H5) environmental contamination: market type (p = 0.036) and location of poultry slaughtering (p = 0.014) in DNCC markets and weekly market closure of >1 day (p = 0.006) in DSCC markets (Appendix Table 2).

### Factors Associated with Influenza A(H5) Virus Environmental Contamination within LBMs

#### Factors Associated with the Probability of LBM Influenza A(H5) Environmental Contamination Risk

We demonstrated by univariable analysis that the probability of influenza A(H5) environmental contamination was significantly higher in slaughtering and processing zones (relative risk [RR] 1.22, 95% CI 1.01–1.49; p = 0.041) than in market arrival zones. The probability of contamination was significantly higher in March (RR 1.90, 95% CI 1.36–2.65; p≤0.001) than January (Table 1).

**Table 1:** Risk factors associated with the probability of influenza A(H5) environmental contamination at specific live bird market work zones, Dhaka, Bangladesh, January–March 2016

Risk factor	Univariable analysis				Multivariable model 1				Multivariable model 2		
	RR (95% CI)	p value	Overall p value		RR (95% CI)	p value	Overall p value		RR (95% CI)	p value	Overall p value
Market work zones of sample collection; reference: arrival											
Slaughtering and processing	1.22 (1.01–1.49)	0.041	0.110		1.23 (1.01–1.50)	0.040	0.103		1.21 (0.99–1.49)	0.067	0.180
Consumer exposure or sales	1.05 (0.84–1.31)	0.647			1.05 (0.84–1.32)	0.655			1.09 (0.86–1.37)	0.487	
Month of sample collection; reference: January											
February	1.24 (0.87–1.77)	0.233	<0.001		1.24 (0.87–1.76)	0.239	<0.001		1.33 (0.91–1.94)	0.138	<0.001
March	1.90 (1.36–2.65)	<0.001			1.90 (1.36–2.65)	<0.001			2.07 (1.44–2.96)	<0.001	
Market type; reference: dual-purpose†											
Wholesale	0.79 (0.57–1.10)	0.161	0.042						0.79 (0.571.10)	0.161	0.042
Retail	0.69 (0.51–0.92)	0.012							0.69 (0.510.93)	0.012	
Species being sold (reference: multiple species)†	0.57 (0.30–1.08)	0.084									
Electricity in facility†	1.50 (0.87–2.60)	0.148									
Market sells poultry to other vendors†	1.21 (0.92–1.58)	0.176									
Weekly market closure (>1 day)†	0.79 (0.55–1.14)	0.207									
Akaike information criterion					1,020.588				932.9017		

*Blank cells indicate variables not included in model. RR, relative risk. †Univariable results adjusted for month of sample collection and market work zones of sample collection.

In the final multivariable analysis (model 2), after adjusting for market-level biosecurity factors, we demonstrated that the probability of influenza A(H5) environmental contamination remained 2-fold significantly higher in March than January (RR 2.07, 95% CI 1.44–2.96; p<0.001). Our findings also demonstrated that slaughtering and processing zones had an increased risk for influenza A(H5) recovery compared to the arrival zone, but this effect was not statistically significant (RR 1.21, 95% CI 0.99–1.49; p = 0.067). In addition, the probability of influenza A(H5) environmental contamination was significantly associated with market type: retail markets were at lower risk than dual-purpose markets (RR 0.69, 95% CI 0.51–0.93; p = 0.012) (Table 1). Model 2 presented a better fit to the data than model 1 (i.e., without adjusting for market-level biosecurity factors). The AIC of model 1 was 1020.6 and in model 2 was 932.9. Effect modification and confounding were not found among pairs of biologically plausible LBM predictor variables.

#### Factors Associated with Work Zone–Specific Influenza A(H5) Virus Environmental Contamination Patterns

Our univariable and multivariable model of the multinomial analysis showed a significant increased risk in all LBM work zone–specific influenza A(H5) environmental contamination patterns except “slaughtering and processing zone area only” in March (relative risk ratio [RRR] >1; null value not contained within 95% CI) compared with January (Table 2). After multivariable adjustment, no market-level factors were significantly associated with work zone–specific influenza A(H5) virus environmental contamination patterns.

**Table 2:** Risk factors associated with live bird market work zone–specific influenza A (H5) virus environmental contamination patterns, Dhaka, Bangladesh, January–March 2016*

Model and selected market-level factors	ASE–positive		S only–positive			SE- or AS-positive			Other positive		AIC
RRR	p value	RRR	p value		RRR	p value		RRR	p value
Univariable model											
Month of sample collection; reference: January											758.5424
February	7.37 (1.93–28.24)	0.004	0.49 (0.15–1.60)	0.237		0.41 (0.14–1.18)	0.098		0.55 (0.22–1.36)	0.197	
March	10.14 (2.09–49.30)	0.004	2.03 (0.80–5.12)	0.134		3.38 (1.34–8.53)	0.010		2.30 (1.01–5.27)	0.048	
Multivariable model											
Month of sample collection; reference: January											727.5063
February	7.89 (1.92–32.39)	0.004	0.55 (0.16–1.94)	0.356		0.44 (0.14–1.41)	0.168		0.60 (0.24–1.52)	0.282	
March	11.14 (2.16–57.57)	0.004	2.22 (0.81–6.10)	0.121		4.46 (1.59–12.48)	0.004		2.55 (1.05–6.19)	0.038	
Total no. poultry head in the market; reference: >1,000											
501–1,000	1.23 (0.33–4.68)	0.757	0.54 (0.15–1.92)	0.338		0.56 (0.19–1.65)	0.295		0.50 (0.16–1.56)	0.233	
1–500	0.87 (0.24–3.19)	0.830	1.55 (0.60–3.98)	0.363		0.58 (0.19–1.73)	0.328		1.07 (0.45–2.54)	0.880	
Weekly market closure >1 day	0.35 (0.09–1.43)	0.145	0.56 (0.20–1.57)	0.274		1.08 (0.48–2.41)	0.850		0.53 (0.22–1.25)	0.144	
Presence of roof	2.75 (0.59–12.75)	0.196	0.55 (0.20–1.54)	0.257		0.72 (0.20–2.57)	0.613		0.55 (0.25–1.20)	0.133	
Sale of poultry to other vendors	1.05 (0.31–3.54)	0.933	2.26 (0.94–5.43)	0.069		1.33 (0.53–3.37)	0.542		1.38 (0.58–3.31)	0.470	
Sale of products other than poultry (e.g., fish, red meat)	2.96 (0.67–13.16)	0.153	0.93 (0.38–2.27)	0.872		0.82 (0.32–2.10)	0.681		0.90 (0.40–2.02)	0.793	
Market location; reference: DSCC	1.34 (0.44–4.03)	0.605	1.06 (0.42–2.64)	0.901		0.87 (0.38–2.00)	0.749		0.93 (0.43–2.01)	0.855	

*A, arrival zone; AIC, Akaike information criterion; DSCC, Dhaka South City Corporation; E, consumer exposure or sales zone; RRR, relative risk ratio; S, slaughtering and processing zone.

## Discussion

Our analyses provide the most comprehensive account of the recovery of influenza A(H5) virus in specific LBM work zones over 3 months across a large sample of LBMs (n = 104) within the Dhaka metropolitan area of Bangladesh. This study overcomes many of the limitations seen in previous studies of LBMs in Dhaka in the context of within-market measurement of environmental contamination (*11*,*12*,*19*,*20*,*22*,*25*).

Our descriptive results indicated vulnerabilities in LBMs in Dhaka associated with increased proportions of influenza A(H5) virus environmental contamination. Previous studies have shown that dual-purpose LBMs (i.e., markets conducting both wholesale and retail operations) in Dhaka were at higher risk for influenza A contamination (*11*). This previous finding suggests that markets in DNCC would be at greater risk for influenza A(H5) contamination. Our analyses confirmed this suggestion, demonstrating a larger proportion of influenza A(H5) recovery in dual-purpose DNCC markets than in retail-only markets. Poultry slaughtering has been consistently found to be a significant risk factor for LBM environmental contamination with influenza A(H5), and studies in Indonesia (*2*,*27*) and Bangladesh (*19*) support this observation. Environmental contamination with influenza A(H5) was significantly higher in DNCC markets without slaughtering facilities than in those reporting poultry slaughtering. Market environmental contamination in the absence of slaughtering facilities could be linked to the sampling procedure, in which sample collectors were instructed to use their sense of perceived risk if suggested sampling sites were not present in the market and other sites had to be chosen. This limitation in the sampling procedure should be corrected in future studies. Biosecurity practices such as cleaning and market closures have been reported to reduce environmental contamination in LBMs and eliminate risk for human infection with influenza A (*39*). Our results indicate that DSCC markets would benefit from higher rates of closures; a higher proportion of influenza A(H5) contamination was found in DSCC markets that did not perform market closures. In 2017, China established the 1110 policy, which involves daily cleaning, weekly disinfection, monthly closure, and no overnight stay of poultry (*40*). This approach has been successful at reducing the level of contamination within LBMs. This suggests that the implementation of a 1110-type policy in Dhaka’s LBMs would strengthen LBM biosecurity, thereby reducing the level of influenza A(H5) contamination. Taken together, the observed differences in environmental contamination between markets in DSCC and DNCC can partly be explained by poultry slaughter and market management activities and less so by trader and poultry demographics.

Risk for influenza A(H5) infection in humans and poultry has been shown to be associated with movement of live poultry during national festive periods (*41*–*43*). In Bangladesh, demand for poultry products is influenced by traditional customs and rituals, including religious and cultural festivals (*44*–*46*). Our analysis found a 2-fold increase in the probability of environmental contamination in March compared with January, and market-level covariates did not modify this effect. Our analysis indicates the increased probability of influenza A(H5) environmental contamination in March in urban LBMs of Dhaka is likely related to the Bangla new year festival, which occurs in April and is linked to increased demand for poultry products in urban Dhaka LBMs.

We demonstrated that influenza A(H5) environmental contamination was positively associated with 2 market-level covariates: work zone (slaughtering and processing zone compared with arrival zone) and type of market (dual-purpose markets compared with retail-only markets). The higher probability of influenza A(H5) environmental contamination in the slaughtering and processing zone and in dual-purpose markets could be related to the challenge of maintaining adequate sanitation in LBMs with these characteristics. The risk for environmental contamination is known to be increased when slaughtering equipment is not frequently cleaned throughout the day using adequate disinfection protocols (*47*). Market attributes such as the presence of wholesalers in the market (*11*) and within-market trade of asymptomatic poultry between wholesalers and retailers (*44*) explain the higher levels of influenza A(H5) environmental contamination in dual-purpose markets compared with retail markets. Our analysis uncovered biosecurity characteristics that could partially explain these higher levels of influenza A(H5) environmental contamination. For example, dual-purpose markets have greater heterogeneity in poultry species than retail-only markets (Appendix Table 3), which could promote virus introduction. Furthermore, our data suggest that the *Sonali* chicken crossbreed was dominant in dual-purpose markets compared with other markets (Appendix Table 3); this crossbreed has previously been shown to have a higher bird-level influenza A(H5) prevalence (*11*).

Our study revealed a significantly increased probability of influenza A(H5) environmental contamination in March in 3 of the 4 site-specific influenza A(H5) environmental contamination patterns. Our results also extend those from a recent study by demonstrating that, outside the month of March, the slaughter area was the environmental site most contaminated with influenza A(H5) in LBMs (*25*). Our findings suggest that to increase the probability of detection of influenza A(H5) environmental contamination, those conducting surveillance should consider the slaughtering and processing zone as the candidate sampling site within LBMs during the months leading up to the increased demand for poultry in April. Furthermore, our results suggest that market-level biosecurity characteristics did not influence the temporal variation in work zone–specific influenza A(H5) environmental contamination patterns (Appendix Figure 1).

Of note, only 1 market-level characteristic (market sells poultry to other traders) was reported to be marginally associated with the probability of S-only environmental contamination pattern. This relationship could be partly explained by the fact that LBM contamination level is not simply the result of continuous introductions of infected birds, but a consequence of virus recirculation and amplification within them (*1*). To further elucidate the market work zone–specific influenza A(H5) environmental contamination patterns identified in this study, follow-up studies into the social network of poultry trade in LBMs are needed to clarify the effect.

The first limitation of our study is that, although we triangulated information on Dhaka LBM characteristics from data collectors with that from market managers through telephone call data validation, the use of secondary data might have introduced undue reporting bias. Second, we focused our analyses on the 3-month period of the winter season (January–March); further analyses should consider expanding the temporal scope of the investigation to better understand the seasonal trends identified in this study. Third, we used a sample pooling strategy (i.e., 18-swab pools collected in 5 mL of viral transport medium), which has not been validated for the presence of serial dilution effect and should be evaluated in future studies. However, despite the 18-swab pooling, we found a significant positivity rate in pooled samples. Fourth, because of budgetary limitations, our study was only conducted in LBMs in the Dhaka metropolitan area without consideration of other cities in Bangladesh. Thus, caution should be taken in interpretation, because the environmental contamination of LBMs in Dhaka might not reflect the local idiosyncrasies of LBMs in other cities in Bangladesh. Finally, despite our efforts to address confounding effects, we could not consider other factors that could be associated with contamination levels, including the poultry trade network between LBMs and source farms and the presence of other infection reservoirs in LBMs.

In conclusion, this study demonstrates that LBMs located in DNCC of Dhaka are qualitatively more vulnerable to influenza A(H5) virus environmental contamination. The probability of influenza A(H5) environmental contamination is equally likely across all within-LBM sites investigated and particularly higher in the month of March. The slaughtering and processing zones of LBMs could serve as candidate zones for active surveillance programs. Future work also should evaluate the effects of poultry movement and LBM biosecurity in the epidemiology of influenza A (H5) virus. Sanitation practices, market closures, and slaughtering and processing practice interventions within LBMs would help to reduce market-level influenza A contamination.

Appendix Additional information about risk areas for influenza A(H5) environmental contamination in live bird markets, Dhaka, Bangladesh
