# Supplementary material for: Risk Areas for Influenza A(H5) Environmental Contamination in Live Bird Markets, Dhaka, Bangladesh
Source: Emerg Infect Dis. 2021 Sep;27(9):2399–408. doi: 10.3201/eid2709.204447 (PMC8386803; doi:10.3201/eid2709.204447)
Supplement: Appendix — Additional information about risk areas for influenza A(H5) environmental contamination in live bird markets, Dhaka, Bangladesh [file 20-4447-Techapp-s1.pdf]

Article DOI: https://doi.org/10.3201/eid2709.204447

Risk Areas for Influenza A (H5) Environmental Contamination in Live Bird Markets, Dhaka, Bangladesh

Appendix

The Rationale of Live Bird Market Selection

In collaboration with the Department of Livestock Services (DLS) in Bangladesh, 2015 data from earlier projects on the presence of live bird markets (LBMs) in Dhaka were made available to the in Bangladeshi office of the Emergency Centre for Transboundary Animal Diseases (ECTAD) of the Food and Agriculture Organization of the United Nations (FAO-UN). LBM data from these earlier projects were merged and duplicate LBMs were identified and removed, yielding a total of 230 LBMs in Dhaka. Of the 230 LBMs, 110 had location information available; influenza surveillance ("AI Sink Surveillance" in LBMs) was initiated in January 2016 in these 110 LBMs to detect influenza A (H5) virus contamination (M.G. Osmani, FAO Bangladesh and Department of Livestock Services, pers. comm., 2016 Nov). In our study, we included 104 markets (of the 110 markets surveilled) in which influenza A (H5) environmental contamination was laboratory-confirmed (*1*). All 104 markets targeted for sampling were visited multiple times during the study period (i.e., each market visited once a month) (*1*).

Background of LBM Census and Market-Level Characteristic Selection

A LBM census in Dhaka was designed and carried out by the ECTAD of FAO-UN Bangladesh in collaboration with DLS to develop a comprehensive database of all LBMs in Dhaka that would be freely shared with participating and interested parties. The specific objectives of the census were to collect information on the number and type of poultry, available facilities and location information for all poultry markets in Dhaka; and to develop a database containing all validated LBM data to be made available to collaborators and interested parties.

During January–March 2016, a LBM census in the Dhaka metropolitan area was conducted by veterinary students of the Sher-E-Bangla Agricultural University (SAU) of Dhaka with support from the staff of Dhaka North City Corporation (DNCC) and Dhaka South City Corporation (DSCC). The role of DNCC and DSCC staff was to confirm the location of the market in Dhaka. Markets were identified by walking all roads in a ward (an administrative subdivision unit in the Dhaka city) and asking local traders and consumers if they knew of any poultry sellers in the area. A market was defined as a place in which $\geq$1 traders sell live poultry at least once a week to end-users, other traders, or both. Market management and biosecurity questionnaires were prepared and pretested by Bangladesh FAO-UN ECTAD technical staff by interviewing a small set of market vendors within Dhaka. Students and staff were trained on census standard operating procedures and forms through a 2-day workshop held at SAU. Each LBM was visited once in the census period and data were collected by questionnaires. The census included data collection on the market type (retail, wholesale, or dual-purpose [both wholesale and retail]), poultry-trading statistics (number of vendors and volumes and species of poultry), available facilities (such as running water, electricity, and roof), and biosecurity characteristics (such as daily cleaning protocol, market closure schedule, and poultry slaughtering locations) (FAO Bangladesh and Department of Livestock Services, pers. comm., 2016). The LBM census reported 659 LBMs in the Dhaka metropolitan area; 326 were located in Dhaka North and the remainder in Dhaka South.

In our study, we compared the LBM census database with the infection database (influenza surveillance) of January–March 2016. By comparing market addresses, we identified 97 markets that appeared in both databases. A total of 7 markets from the infection database were not identified through the census. We used the LBM census information (i.e., market-level characteristics) for 97 markets along with market infection data for 104 markets to quantify risk factors associated with the probability of influenza A (H5) virus environmental contamination in specific market work zones, as well as to work zone–specific environmental contamination patterns.

Selection of Environmental Sites in Work Zones within LBMs

A total of 3 environmental work zones within LBM were selected for sampling on the basis of a previous study in Indonesia that showed significant likelihood of influenza A (H5) contamination (*2*). These zones were sampled once a month during the January–March 2016

study period. Although the environmental work zones were specific, the location of environmental sampling sites per LBM work zone were not always the same at every market follow-up.

The environmental sampling sites were indicated by work zone. For the poultry arrival zone (A), these sites were sampled: swab 1, floor of arrival/holding area; swab 2, cages or pens with live birds; swab 3, waste water; swab 4, waste bins; swab 5, trucks (if present); swab 6, any one of the above (on the basis of perceived risk). For the poultry slaughtering and processing zone (S), these sites were sampled: swab 1, processing table after defeathering; swab 2, baskets or trays holding poultry meat; swab 3, slaughtering boards/area; swab 4, waste bins; swab 5, waste water/blood drain path; swab 6, any one of the above (on the basis of perceived risk). For the consumer exposure or sales zone (E), these sites were sampled: swab 1, table for display; swab 2, chopping boards; swab 3, wet cleaning cloths; swab 4, scales; swab 5, knives/utensils; swab 6, any one of the above (on the basis of perceived risk).

Multivariable Statistical Modeling of LBM–Level Influenza A (H5) Environmental Contamination Risk

To quantify risk factors associated with the probability of influenza A (H5) virus environmental contamination in specific LBM work zones (i.e., arrival, slaughtering and processing, consumer exposure or sales), we performed our analysis in 3 steps. First, we developed univariable Bernoulli generalized linear models by using a log link function and a market random effect to screen variables with $p<0.20$ to be considered in a full multivariable model. Second, before inclusion in the full multivariable model, correlations between selected variables were investigated by using tetrachoric and polychoric correlation methods for binary and ordered-category variables (*3*). The purpose of checking correlation was to reduce or avoid collinearity among predictor variables that can lead to biased estimates and inflated standard errors (*4*). If 2 predictors were highly correlated (correlation coefficient >0.70) (*5*), only 1 of the variables was included in the full multivariable model. Third, we established 2 multivariable models: a multivariable model to understand the effects of month of sample collection and LBM work zone on the probability of environmental contamination risk (model 1), and a multivariable model to understand how market-level factors influence the effect of month of sample collection and LBM work zone level on the probability of environmental contamination risk (model 2). We arrived at the final multivariable model by using a backward elimination variable selection

process and retained predictors significant at $p<0.05$ (all reported p values are 2-sided) and those that could be deemed confounders. Confounding was checked by adding and removing a variable from the model and assessing the impact on coefficients of other variables; if the change was >25%, that variable was deemed a confounder and was retained in the final multivariable model. Effect modification was also investigated for pairs of predictor variables on the basis of biologic plausibility. Generalized joint Wald tests were used to test the significance of each fitted categorical variable with >2 levels. Akaike information criterion (AIC) was used to determine the best-fitting multivariable model (*6*). The best-fitting model was the one with the lowest AIC among all competing models (*6*).

To quantify the association between LBM work zone–specific influenza A (H5) environmental contamination patterns and the month of sample collection, we developed univariable and multivariable multinomial logistic regression models, which included a random effect of market to account for repeated measurements at the market level. The univariable model only included the month of sample collection (i.e., month categorized into January, February, and March). The multivariable model was further adjusted for market-level factors to assess the influence of these factors on the relationship between LBM work zone–specific influenza A (H5) environmental contamination patterns and timing of sampling. We arrived at the final and best-fitting multivariable multinomial model following the process outlined above for the Bernoulli generalized linear model without backward elimination variable selection process. Factors with insufficient data across different categories were not considered in the final multivariable model analysis.

We have used a Bernoulli family logistic regression to model the error in the probability of influenza A (H5) recovery as opposed to a binomial logistic regression model specification. Retaining this nomenclature is better to reflect the nature of the model used and will also help differentiate this model from the additional multinomial logistic regression model we developed to provide insight into site-specific infection patterns.

References

1. Osmani M, Akwar H, Hasan Z, Chakma S, Hossain MM, Brum E. Sink surveillance, an innovative approach to identify HPAI and other emerging zoonotic pathogens in live bird markets in Bangladesh. Prince Mahidol Award Conference (PMAC); Bangkok, Thailand; 2018.

2. Indriani R, Samaan G, Gultom A, Loth L, Irianti S, Adjid R, et al. Environmental sampling for avian influenza virus A (H5N1) in live-bird markets, Indonesia. Emerg Infect Dis. 2010;16:1889–95. PubMed https://doi.org/10.3201/eid1612.100402

3. Juras J, Pasarić Z. Application of tetrachoric and polychoric correlation coefficients to forecast verification. Geofizika. 2006;23:59–82.

4. Alexopoulos EC. Introduction to multivariate regression analysis. Hippokratia. 2010;14(Suppl 1):23–8. PubMed

5. Dormann CF, Elith J, Bacher S, Buchmann C, Carl G, Carré G, et al. Collinearity: a review of methods to deal with it and a simulation study evaluating their performance. Ecography. 2013;36:27–46. https://doi.org/10.1111/j.1600-0587.2012.07348.x

6. Mohammed EA, Naugler C, Far BH. Emerging business intelligence framework for a clinical laboratory through big data analytics. In: Arabnia H, Tran QN, editors. Emerging trends in computational biology, bioinformatics, and systems biology. Amsterdam: Elsevier; 2015. p. 577–602.

Appendix Table 1. Characteristics of live bird markets in Dhaka metropolitan area, Bangladesh, January–March 2016*

	No. (%)		
Market level characteristics	Dhaka area (DSCC and DNCC)	DSCC	DNCC
Market type			
Dual purpose (wholesale and retail)	22 (22.68)	6 (11.54)	16 (35.56)
Wholesale	2 (2.06)	2 (3.85)	0
Retail	73 (75.26)	44 (84.61)	29 (64.44)
Species being sold			
Multiple	90 (92.78)	46 (88.46)	44 (97.78)
Single	7 (7.22)	6 (11.54)	1 (2.22)
No. vendors			
>15	23 (23.71)	12 (23.07)	11 (24.44)
11–15	16 (16.49)	9 (17.31)	7 (15.56)
6–10	23 (23.71)	9 (17.31)	14 (31.11)
1–5	35 (36.08)	22 (42.31)	13 (28.89)
No. species being sold			
7–9	8 (8.25)	1 (1.92)	7 (15.56)
4–6	59 (60.82)	30 (57.69)	29 (64.44)
1–3	30 (30.93)	21 (40.39)	9 (20.00)
Dominant species (by comparing poultry headcount)			
Broiler	72 (74.23)	36 (69.24)	36 (80.00)
Deshi	9 (9.28)	8 (15.38)	1 (2.22)
Sonali	16 (16.49)	8 (15.38)	8 (17.78)
Number of poultry head			
>1,000	39 (40.21)	18 (34.61)	21 (46.66)
501–1,000	24 (24.74)	12 (23.08)	12 (26.67)
1–500	34 (35.05)	22 (42.31)	12 (26.67
Electricity in facility			
No	7 (7.22)	4 (7.69)	3 (6.67)
Yes	90 (92.78)	48 (92.31)	42 (93.33)
Presence of roof			
No	16 (16.49)	8 (15.38)	8 (17.78)
Yes	81 (83.51)	44 (84.62)	37 (82.22)
Running water in facility			
No	45 (46.39)	22 (42.31)	23 (51.11)
Yes	52 (53.61)	30 (57.69)	22 (48.89)

	No. (%)		
Market level characteristics	Dhaka area (DSCC and DNCC)	DSCC	DNCC
Sell poultry to other vendors			
No	69 (71.13)	41 (78.85)	28 (62.22)
Yes	28 (28.87)	11 (21.15)	17 (37.78)
Weekly market closure (≥1 day)			
No	65 (67.01)	28 (53.85)	37 (82.22)
Yes	32 (32.99)	24 (46.15)	8 (17.78)
Sell poultry to consumers directly			
No	4 (4.12)	2 (3.85)	2 (4.44)
Yes	93 (95.88)	50 (96.15)	43 (95.56)
Sell products other than poultry (e.g., fish, red meat, vegetables, groceries)			
No	21 (21.65)	17 (32.69)	4 (8.89)
Yes	76 (78.35)	35 (67.31)	41 (91.11)
Daily cleaning protocol (at minimum with detergent)			
No	35 (36.08)	13 (25.00)	22 (48.89)
Yes	62 (63.92)	39 (75.00)	23 (51.11)
Poultry slaughtering location			
Vendor stall	83 (85.57)	41 (78.85)	42 (93.34)
Central facility	2 (2.06)	1 (1.92)	1 (2.22)
Vendor stall and central facility	4 (4.12)	3 (5.77)	1 (2.22)
Vendor stall and outside market	4 (4.12)	4 (7.69)	0
No facility	4 (4.12)	3 (5.77)	1 (2.22)
No. slaughtering facilities			
>10	31 (31.96)	17 (32.69)	14 (31.11)
6–10	26 (26.8)	10 (19.23)	16 (35.56)
0–5	40 (41.24)	25 (48.08)	15 (33.33)

*DNCC, Dhaka North City Corporation; DSCC, Dhaka South City Corporation.

Appendix Table 2. Differences in the proportion of influenza A (H5) virus environmental contamination in live bird markets, Dhaka metropolitan area, January–March 2016*

	Dhaka area (DSCC and DNCC)			DSCC			DNCC		
Selected market-level factors	N	No. (%)	p value†	N	No. (%)	p value†	N	No. (%)	p value†
Month of sample collection									
January	297	62 (20.88)	<0.001	153	32 (20.92)	0.049	144	30 (20.83)	<0.001
February	282	73 (25.89)		141	35 (24.82)		141	38 (26.95)	
March	288	114 (39.58)		144	48 (33.33)		144	66 (45.83)	
Market work zone									
Arrival	289	76 (26.30)	0.268	146	35 (23.97)	0.665	143	41 (28.67)	0.401
Slaughtering and processing	289	93 (32.18)		146	42 (28.77)		143	51 (35.66)	
Consumer exposure or sales	289	80 (27.68)		146	38 (26.03)		143	42 (29.37)	
Market type									
Dual-purpose (wholesale and retail)	174	66 (37.93)	0.006	42	16 (38.10)	0.150	132	50 (37.88)	0.036
Wholesale	18	5 (27.78)		18	5 (27.78)		0	0 (0.00)	
Retail	615	157 (25.53)		369	90 (24.39)		246	67 (27.24)	
Weekly market closure (≥1 day)									
No	552	168 (30.43)	0.044	237	74 (31.22)	0.006	315	94 (29.84)	0.300
Yes	255	60 (23.53)		192	37 (19.27)		63	23 (36.51)	
Species being sold									
Multiple	744	217 (29.17)	0.057	375	102 (27.20)	0.134	369	115 (31.17)	0.727
Single	63	11 (17.46)		54	9 (16.67)		9	2 (22.22)	
Electricity in facility									
No	54	10 (18.52)	0.118	33	6 (18.18)	0.408	21	4 (19.05)	0.331
Yes	753	218 (28.95)		396	105 (26.52)		357	113 (31.65)	
Sell poultry to consumers directly									
No	36	9 (25.00)	0.850	18	5 (27.78)	0.789	18	4 (22.22)	0.602
Yes	771	219 (28.40)		411	106 (25.79)		360	113 (31.39)	
Sell poultry to other vendors									
No	573	153 (26.70)	0.143	336	81 (24.11)	0.141	237	72 (30.38)	0.818
Yes	234	75 (32.05)		93	30 (32.26)		141	45 (31.91)	
Sale of products other than poultry (e.g., fish, red meat, vegetables, groceries)									
No	183	46 (25.14)	0.306	147	36 (24.49)	0.728	36	10 (27.78)	0.850
Yes	624	182 (29.17)		282	75 (26.60)		342	107 (31.29)	
Presence of roof									
No	135	36 (26.67)	0.677	69	19 (27.54)	0.765	66	17 (25.76)	0.380
Yes	672	192 (28.57)		360	92 (25.56)		312	100 (32.05)	
Running water in facility									
No	384	111 (28.91)	0.696	192	55 (28.65)	0.268	192	56 (29.17)	0.505
Yes	423	117 (27.66)		237	56 (23.63)		186	61 (32.80)	
Daily cleaning protocol (at minimum with detergent)									
No	294	82 (27.89)	0.871	114	26 (22.81)	0.454	180	56 (31.11)	1.000
Yes	513	146 (28.46)		315	85 (26.98)		198	61 (30.81)	
Poultry slaughtering locations									
Vendor stall only	699	192 (27.47)	0.171	345	87 (25.22)	0.599	354	105 (29.66)	0.014
Other locations (central facility only, vendor stall, and other sites)	75	22 (29.33)		57	15 (26.32)		18	7 (38.89)	
No slaughtering facility	33	14 (42.42)		27	9 (33.33)		6	5 (83.33)	
No. slaughtering facilities									

Selected market-level factors	Dhaka area (DSCC and DNCC) N	No. (%)	p value†	DSCC N	No. (%)	p value†	DNCC N	No. (%)	p value†
>10	258	83 (32.17)	0.231	138	43 (31.16)	0.163	120	40 (33.33)	0.714
6–10	210	57 (27.14)		72	14 (19.44)		138	43 (31.16)	
0–5	339	88 (25.96)		219	54 (24.66)		120	34 (28.33)	
No. vendors									
>15	195	56 (28.72)	0.642	102	30 (29.41)	0.166	93	26 (27.96)	0.900
11–15	129	42 (32.56)		69	22 (31.88)		60	20 (33.33)	
6–10	186	49 (26.34)		66	11 (16.67)		120	38 (31.67)	
1–5	297	81 (27.27)		192	48 (25.00)		105	33 (31.43)	
No. species being sold									
7–9	66	20 (30.30)	0.656	9	1 (11.11)	0.617	57	19 (33.33)	0.866
4–6	489	142 (29.04)		237	64 (27.00)		252	78 (30.95)	
1–3	252	66 (26.19)		183	46 (25.14)		69	20 (28.99)	
Dominant species (by comparing poultry headcount)									
Broiler	606	173 (28.55)	0.696	303	76 (25.08)	0.766	303	97 (32.01)	0.266
Deshi	60	14 (23.33)		54	14 (25.93)		6	0 (0.00)	
Sonali	141	41 (29.08)		72	21 (29.17)		69	20 (28.99)	
No. poultry head									
>1,000	327	103 (31.50)	0.239	144	46 (31.94)	0.068	183	57 (31.15)	0.549
501–1,000	195	52 (26.67)		96	18 (18.75)		99	34 (34.34)	
1–500	285	73 (25.61)		189	47 (24.87)		96	26 (27.08)	

*DNCC, Dhaka North City Corporation; DSCC, Dhaka South City Corporation.
†p values by Fisher's exact test.

Appendix Table 3. Characteristics of live bird markets by market types in Dhaka metropolitan area, Bangladesh, January–March, 2016*

Market-level characteristics	No. (%) Dual-purpose (wholesale and retail)	Wholesale	Retail	Total	p value†
No. vendors					
>15	10 (43.48)	2 (8.70)	11 (47.83)	23	0.006
11–15	4 (25.00)	0	12 (75.00)	16	
6–10	5 (21.74)	0	18 (78.26)	23	
1–5	3 (8.57)	0	32 (91.43)	35	
Sell poultry to consumers directly					
No	1 (25.00)	2 (50.00)	1 (25.00)	4	0.001
Yes	21 (22.58)	0	72 (77.42)	93	
Sell poultry to other vendors					
No	5 (7.25)	0	64 (92.75)	69	<0.001
Yes	17 (60.71)	2 (7.14)	9 (32.14)	28	
Sell products other than poultry (e.g., fish, red meat, vegetables, groceries)					
No	3 (14.29)	1 (4.76)	17 (80.95)	21	0.266
Yes	19 (25.00)	1 (1.32)	56 (73.68)	76	
Running water in facility					
No	11 (24.44)	1 (2.22)	33 (73.33)	45	0.904
Yes	11 (21.15)	1 (1.92)	40 (76.92)	52	
Electricity in facility					
No	0	0	7 (100.00)	7	0.308
Yes	22 (24.44)	2 (2.22)	66 (73.33)	90	
Presence of roof					
No	2 (12.50)	0	14 (87.50)	16	0.545
Yes	20 (24.69)	2 (2.47)	59 (72.84)	81	
Daily cleaning protocol (at minimum with detergent)					
No	8 (22.86)	1 (2.86)	26 (74.29)	35	1.000
Yes	14 (22.58)	1 (1.61)	47 (75.81)	62	
Weekly market closure (≥1 day)					
No	14 (21.54)	1 (1.54)	50 (76.92)	65	0.734
Yes	8 (25.00)	1 (3.13)	23 (71.88)	32	
No. slaughtering facilities					
>10	11 (35.48)	0	20 (64.52)	31	0.036
6–10	7 (26.92)	0	19 (73.08)	26	
0–5	4 (10.00)	2 (5.00)	34 (85.00)	40	
Poultry slaughtering locations					
Vendor stall	18 (21.69)	0	65 (78.31)	83	0.001
Central facility	2 (100.00)	0	0 (0.00)	2	
Vendor stall and central facility	1 (25.00)	0	3 (75.00)	4	
Vendor stall and outside market	0	0	4 (100.00)	4	
No facility	1 (25.00)	2 (50.00)	1 (25.00)	4	
Species being sold					
Multiple	22 (24.44)	2 (2.22)	66 (73.33)	90	0.308
Single	0	0	7 (100.00)	7	
No. poultry species being sold					
7–9	5 (62.50)	0	3 (37.50)	8	0.002
4–6	15 (25.42)	0	44 (74.58)	59	
1–3	2 (6.67)	2 (6.67)	26 (86.67)	30	
Dominant species (by comparing poultry headcount)					
Broiler	12 (16.67)	1 (1.39)	59 (81.94)	72	0.018
Deshi	2 (22.22)	0	7 (77.78)	9	
Sonali	8 (50.00)	1 (6.25)	7 (43.75)	16	
Market location					
DSCC	6 (11.54)	2 (3.85)	44 (84.62)	52	0.005
DNCC	16 (35.56)	0	29 (64.44)	45	
Total no. poultry head					
>1000	14 (35.90)	2 (5.13)	23 (58.97)	39	0.004
501–1000	6 (25.00)	0	18 (75.00)	24	
1–500	2 (5.88)	0	32 (94.12)	34	

*DNCC, Dhaka North City Corporation; DSCC, Dhaka South City Corporation.
†p value by Fisher's exact test.

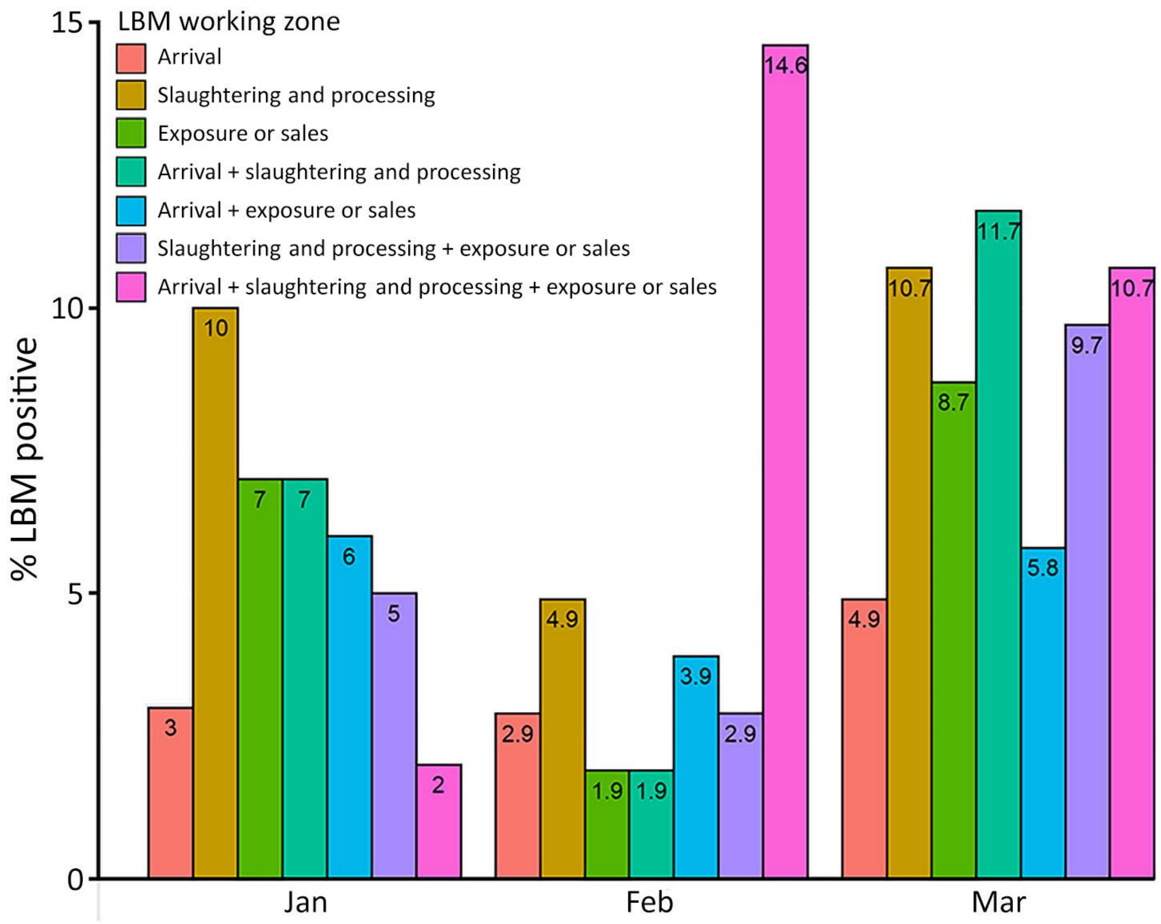

Appendix Figure. Distribution of market work zone–specific influenza A (H5) environmental contamination patterns at live bird markets (LBMs) of Dhaka metropolitan area, Bangladesh, January–March 2016. Exposure or sales refers to consumer exposure.
